# Accuracy and precision in super-resolution MRI: Enabling spherical tensor diffusion encoding at ultra-high b-values and high resolution

**DOI:** 10.1016/j.neuroimage.2021.118673

**Published:** 2021-10-21

**Authors:** Geraline Vis, Markus Nilsson, Carl-Fredrik Westin, Filip Szczepankiewicz

**Affiliations:** a Department of Diagnostic Radiology, Clinical Sciences Lund, Lund University, Lund, Sweden; b Department of Radiology, Brigham and Women’s Hospital Harvard Medical School, Boston, MA, United States

**Keywords:** Super-resolution reconstruction, Diffusion magnetic resonance imaging, Tensor-valued diffusion encoding, Ultra-high b-values, Rectified noise floor, Noise propagation

## Abstract

Diffusion MRI (dMRI) can probe the tissue microstructure but suffers from low signal-to-noise ratio (SNR) whenever high resolution is combined with high diffusion encoding strengths. Low SNR leads to poor precision as well as poor accuracy of the diffusion-weighted signal; the latter is caused by the rectified noise floor and can be observed as a positive bias in magnitude signal. Super-resolution techniques may facilitate a beneficial tradeoff between bias and resolution by allowing acquisition at low spatial resolution and high SNR, whereafter high spatial resolution is recovered by image reconstruction. In this work, we describe a super-resolution reconstruction framework for dMRI and investigate its performance with respect to signal accuracy and precision. Using phantom experiments and numerical simulations, we show that the super-resolution approach improves accuracy by facilitating a more beneficial trade-off between spatial resolution and diffusion encoding strength before the noise floor affects the signal. By contrast, precision is shown to have a less straightforward dependency on acquisition, reconstruction, and intrinsic tissue parameters. Indeed, we find a gain in precision from super-resolution reconstruction is substantial only when some spatial resolution is sacrificed. Finally, we deployed super-resolution reconstruction in a healthy brain for the challenging combination of spherical b-tensor encoding at ultra-high b-values and high spatial resolution—a configuration that produces a unique contrast that emphasizes tissue in which diffusion is restricted in all directions. This demonstration showcased that super-resolution reconstruction enables a vastly superior image contrast compared to conventional imaging, facilitating investigations that would otherwise have prohibitively low SNR, resolution or require non-conventional MRI hardware.

## 1. Introduction

Diffusion MRI (dMRI) is a non-invasive method for investigating tissue microstructure in healthy and pathological tissue. Investigations of subtle microstructure features rely on the use of strong diffusion-weighting (ultra-high b-values) or tensor-valued diffusion encoding (Jensen et al., 2005; Tax et al., 2020; Westin et al., 2016), both of which frequently suffer from low signal-to-noise ratios (SNR). Low SNR may cause poor signal accuracy in magnitude imaging due to the so-called rectified noise floor, which causes a positive signal bias (Gudbjartsson and Patz, 1995). Unlike precision, signal accuracy is not improved by averaging over magnitude signals unless the phase is coherent across the observations (Jones and Basser, 2004). This is challenging because the diffusion encoding causes phase variation in the presence of tissue motion (Eichner et al., 2015; Prah et al., 2010). Therefore, it is challenging to perform accurate measurements of diffusion-weighted signal at low SNR.

In addition to strong diffusion-weighting, an approach that regularly suffers from low SNR is spherical or isotropic b-tensor encoding, especially at ultra-high b-values. Although high b-value spherical encoding is challenging, it is desirable because it emphasizes signal from tissue in which diffusion is restricted along all directions. For example, it can be used to highlight the tightly packed granule cells in the cerebellar cortex, which are affected in diseases such as spinocerebellar ataxis and Alzheimer disease (Fukutani et al., 1996; Xia et al., 2013). So far, this contrast has been demonstrated at preclinical MRI (Lundell et al., 2019) and MRI systems with ultra-strong gradients and low spatial resolution (Tax et al., 2020). Making this imaging contrast available at clinical MRI scanners and high spatial resolution may enable studies of the cerebellum in a wide range of neurological conditions.

Super-resolution reconstruction (SRR) is a promising solution to the problem of low SNR in dMRI. In SRR, data is acquired at a low spatial resolution—with improved precision and accuracy—and a specialized image reconstruction is used to reconstruct a high-resolution image. Several approaches have been proposed throughout the years, showing SRR methods can balance the trade-off between SNR, spatial resolution and acquisition time (Greenspan, 2009; Greenspan et al., 2002; Jeurissen et al., 2018; Plenge et al., 2012; Poot et al., 2013; Scherrer et al., 2012; Shilling et al., 2009; Souza and Senn, 2008; Van Reeth et al., 2012; Van Steenkiste et al., 2016; Yang et al., 2018). For example, Yang et al. proposed a strategy where joint information from the adjacent scanning directions was used to improve resolution. Poot et al. (2012) demonstrated increased resolution of diffusion tensor parameters from a set of super-resolved diffusion-weighted images with fully sampled diffusion-weightings and gradient directions, while Van Steenkiste et al. (2016) showed increased spatial resolution of diffusion tensor parameters when optimizing both k- and q-space sampling. More recently, Jeurissen et al. (2018) showed an improvement of both accuracy and precision in q-space trajectory imaging parameter estimation, where higher diffusion-weightings are used. These studies also illustrate a flexibility in how the low-resolution data is acquired. For example, the image acquisition can comprise three orthogonal low-resolution axes (Souza and Senn, 2008), slice shifting along the low-resolution direction (Greenspan et al., 2002), or multiple stacks of slices rotated about a common axis (Shilling et al., 2009). This flexibility allows SRR to be tailored to the needs of echo-planar imaging (EPI) which is the imaging readout strategy most used in dMRI. For example, using a common phase encoding direction across the low-resolution acquisitions avoid that local field inhomogeneities cause variable geometric distortions (Jezzard et al., 1998).

Although SRR has seen uptake in several MRI applications, the literature lacks a systematic treatment of noise propagation and how it affects accuracy and precision. For example, imaging at a low resolution allows faster imaging (shorter repetition times), but leads to a complex interplay between SNR, scan time, repetition time, and T1-relaxation. An understanding of these trade-offs is necessary for an optimized experimental design. Furthermore, the application of SRR for spherical b-tensor encoding at ultra-high b-values and high resolution are yet to be explored. In this work, we aim to formally analyze noise propagation in SRR and experimentally verify the impact of SRR on signal accuracy and precision. Finally, we demonstrate the value of SRR in the challenging combination of spherical b-tensor encoding at a b-value of 4.0 ms/μm^2^ and 1.6 mm^3^ isotropic resolution in the brain.

## 2. Theory

Super-resolution reconstruction aims at recovering a high-resolution image from multiple low-resolution images that sample the object in complementary ways. Here we acquired multiple stacks of thick slices rotated around the phase encoding direction (Shilling et al., 2009). To reconstruct an image with isotropic voxels, the lower limit of low-resolution rotations *N*_*R*_ is given by (Plenge et al., 2012)

(1) $$N_{R}\geq \frac{\pi }{2}\cdot \alpha ,$$

where α is the aspect factor, i.e., the ratio between the long and short axis of the voxel. Note that this factor also captures the volume ratio between the low and high-resolution voxels, assuming that all other parameters are unchanged, and that the SNR is proportional to the voxel volume in multi-slice acquisitions (Edelstein et al., 1986).

The mapping from a high-resolution image (**x**) to a low resolution image (**y**) can be described by a linear system (Elad and Feuer, 1997)

(2) $$y_{k}=A_{k}x+\varepsilon_{k},$$

where *k* is an index of the low-resolution image sample and *ε* is random noise. The sampling matrix **A**_*k*_ describes the rotation/translation, down-sampling and blurring of the high-resolution image. As these factors are known, **A**_*k*_ can be constructed from the image dimensions, voxel spacing and slice profile. Here, we used a box function to describe the slice profile. Both **y**_*k*_ and **x** are expressed as column vectors such that a square image with *N* voxels on each side is represented by a *N* ^2^ × 1 vector. The complete sampling of all low-resolution images can be described in a single linear system

(3) y=Ax+ε.

Assuming the noise is independent and normally distributed with zero mean, the solution to recover **x** given **A** and **y** can be expressed as a least-squares problem, such that

(4) $$\hat{x}=\underset{x}{\text{argmin}}∥Ax-y{∥}_{2}^{2}.$$

The greater the number of complementary observations in **y**, the better the condition of the problem becomes. However, the problem in Eq. (4) often remains ill-posed due to the down-sampling operation included in **A**. There are several ways of solving the reconstruction problem, as thoroughly described in (Plenge et al., 2012). The solution requires some form of regularization, which often translates to imposing a smoothness to the solution (Van Reeth et al., 2012). A common approach is Tikhonov regularization (Tikhonov and Arsenin, 1977), that penalizes high spatial-frequencies in the estimated high-resolution image. Including this regularization, the regularized least-squares problem becomes

(5) $$\hat{x}=\underset{x}{\text{argmin}}∥Ax-y{∥}_{2}^{2}+\lambda ∥R(x){∥}_{2}^{2},$$

where **R** is the regularization term and λ is a scalar weight. We will here use a general regularization term independent of the image content: **R**(**x**) = **I**, where **I** is the identity matrix. This enables the use of the closed form solution, according to

(6) $$\hat{x}={\left(A^{\text{T}}A+\lambda I\right)}^{-1}A^{\text{T}}y.$$

**Fig. 1.**
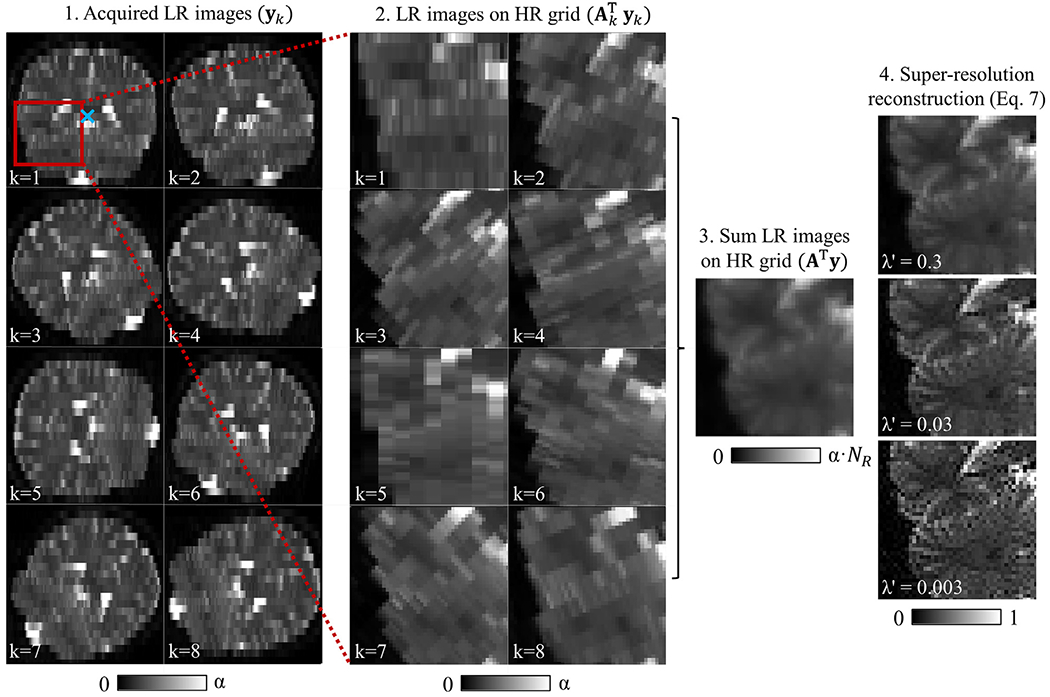
Illustration of the super-resolution reconstruction process in coronal view. Step 1: Multiple images are acquired with low through-plane resolution rotated around the anterior-posterior axis indicated by the blue cross. Step 2: The images are up-sampled to the high-resolution grid by application of the up-sampling operator to each individual image. Step 3: The joint up-sampling operator results in the sum of the individual images, i.e., a smooth image on the high-resolution grid. Step 4: The sharpening operator is applied to finalize the reconstruction process and obtain a high-resolution image. The regularization parameter λ’ determines the trade-off between resolution and noise propagation; a higher λ’ leads to a blurrier, but less noisy image. Steps 2 to 4 are shown as a magnified view of the region indicated by the red square. Color bars show how the intensity scale changes with each step. α is the aspect factor and *N*_*R*_ is the number of low-resolution images.

Note that **A** ^T^ **y** produces the average low-resolution signal on the high-resolution grid, i.e., a blurred image. Without regularization (λ = 0), the remaining term (**A** ^T^ **A**)^−1^ is a sharpening operator, ideally reproducing the true image when applied to **A** ^T^ **y**. As λ increases, the sharpening is reduced. However, as λ alters the denominator in Eq. (6), the intensity scale of $\hat{x}$ is dependent on λ. To remove this dependence, and thereby simplify comparisons among sampling schemes as described later, we can rewrite Eq. (6) according to

(7) $${\hat{x}}^{\prime }={\left(\left(1-\lambda^{\prime }\right)A^{\text{T}}A+\lambda^{\prime }\cdot N_{R}\cdot \alpha \cdot I\right)}^{-1}A^{\text{T}}y=Cy,$$

where **C** contains the entire reconstruction operation. The new regularization factor is constrained to 0 ≤ λ′ ≤ 1, such that λ′ = 1 merely returns the average low-resolution signal on the high-resolution grid, but corrected for the intensity gain caused by larger voxel volumes. Fig. 1 illustrates the SRR process for an in vivo acquisition for weak, moderate, and strong levels of regularization (different values of λ′). Weak regularization amplifies noise, while strong regularization results in a blurred image. Moderate regularization balances the two.

### 2.1. Signal accuracy and precision

As we evaluate the performance of SRR in terms of signal accuracy and signal precision, we will outline the tools used to assess these. Across repeated measurements under identical conditions, signal *accuracy* (or trueness)^1^ concerns the distance between the average signal to the true value, while signal *precision* concerns the spread of the signal. Both terms are influenced by the distribution that characterizes the MR signal. The noise in the complex MR signal is normally distributed, whereas the magnitude signal used in dMRI is approximately Rice distributed (Gudbjartsson and Patz, 1995) (for a detailed review of MR data distributions, see (den Dekker and Sijbers, 2014) or (Veraart et al., 2013)). Consequently, an approximation of the measured signal *M* is given by (Gudbjartsson and Patz, 1995)

(8) $$M=\sqrt{S^{2}+{\left(\sigma \sqrt{\pi /2}\right)}^{2}},$$

where *S* is the signal in the absence of noise and σ is the standard deviation of the noise. In absence of a true signal, the rectified noise floor causes the expected value to be $\sqrt{\pi /2}$. This gives rise to a positive signal bias that becomes prominent at low SNR. Therefore, we employ the signal-to-noise-floor ratio (SNFR) as a measure of overall signal accuracy, defined as (Lätt et al., 2007)

(9) $$\text{SNFR}=\frac{\bar{S}(0)}{\sigma \sqrt{\pi /2}},$$

where $\bar{S}(0)$ is the mean of measured non-diffusion weighted signal over repeated measurements. Note that the SNFR characterizes the maximum achievable signal attenuation for accurate signal sampling. As a measure of precision, we use the signal-to-noise ratio (SNR) defined as

(10) $$\text{SNR}=\frac{\bar{S}(0)}{\sigma }.$$

Note that SNR and SNFR are both proportional to the voxel volume whereas noise levels are independent of the voxel volume (Edelstein et al., 1986).

To enable comparisons of precision between SRR and a conventional high-resolution acquisition (referred to as direct sampling), we define the SNR efficiency factor (ρ), according to

(11) $$\rho =\frac{{\text{SNR}}_{\text{SRR}}}{{\text{SNR}}_{\text{D}}}\cdot \sqrt{\frac{{\text{TA}}_{\text{D}}}{{\text{TA}}_{\text{SRR}}}},$$

where SNR_SRR_ and SNR_D_ are the SNR levels and TA_SRR_ and TA_D_ the acquisition times of SRR and direct sampling at a given spatial resolution. To evaluate Eq. (11), we study how noise propagates from **y** into **x**. For SNR > 3 the noise distribution is approximately Gaussian, independent and identically distributed (Cárdenas-Blanco et al., 2008; Gudbjartsson and Patz, 1995). Under the assumption of identical noise levels among the individual low-resolution images, the signal variance in the reconstructed image can be easily computed from the linear operations in Eq. (7) based on the additive property of variance. We define the noise propagation factor (κ) as the average ratio of the noise standard deviation in the high-resolution reconstructed and low-resolution images, according to

(12) $$\kappa =\frac{1}{n}\overset{n}{\underset{i=1}{\sum^{\text{​}}}}\frac{\sigma_{x}(i)}{\sigma_{y}}=\frac{1}{n}\overset{n}{\underset{i=1}{\sum^{\text{​}}}}\sqrt{\overset{m}{\underset{j=1}{\sum^{\text{​}}}}C{\left(i,j\right)}^{2}}$$

where *n* is the total number of reconstructed voxels in the reconstructed image, indexed by *i*, and *m* is the total number of low-resolution input voxels, indexed by *j*. The noise propagation factor is dependent on properties of the sampling (**A**, *N*_*R*_, α) and the regularization (λ′).

To characterize the impact of SRR on SNR efficiency, we also consider how the signal level changes between a directly sampled and an SRR acquisition. For a setup where the only difference between the two is related to the SRR configuration (slice thickness, number of slices, repetition time and number of slice orientations), Eq. (11) can be extended to include the relevant effects of imaging parameters (Appendix A) to

(13) $$\rho =\frac{1}{\kappa }\cdot \frac{1-\exp\left(-{\text{TR}}_{\text{SRR}}/\text{T}1\right)}{1-\exp\left(-{\text{TR}}_{\text{D}}/\text{T}1\right)}\cdot \sqrt{\frac{{\text{TR}}_{\text{D}}}{N_{R}\cdot {\text{TR}}_{\text{SRR}}}},$$

where TR_D_ and TR_SRR_ are the repetition times for direct sampling and SRR respectively, and T1 is the longitudinal relaxation time. The TR is approximately proportional to the number of slices, and the number of slices to cover the same area in an SRR acquisition can be reduced with a factor of α. Therefore, the minimal TR_SRR_ is given by

(14) $${\text{TR}}_{\text{SRR}}=\frac{{\text{TR}}_{\text{D}}}{\alpha }.$$

When TR_D_ TR_D_ ≫ T1, both 1 − exp(−TR_SRR_/T1) and 1 − exp(−TR_D_/T1)will approach unity, as will their ratio. When sampling directly, approximately 1% signal is lost due to incomplete T1 recovery when TR_D_/T1 = 4.6. However, when using α = 6 and a minimal TR_SRR_, this corresponds to a signal loss of 46%. For this reason, as the aspect factor increases and the TR is minimized, the expected SRR gain will eventually be negated by incomplete T1-recovery.

## 3. Methods

We evaluated the performance of the SRR by three lines of investigation. First, we investigated signal accuracy in a water phantom, both numerically and experimentally. Second, we investigated signal precision in terms of SNR efficiency, both numerically, analytically, and experimentally in vivo. Third, we demonstrated the utility of SRR for spherical b-tensor dMRI at ultra-high b-values in vivo. All simulations and data analysis were performed in Matlab (The MathWorks, Inc., Natick, Massachusetts, USA). The SRR framework is available on https://github.com/filip-szczepankiewicz/Vis_NIMG_2021/.

### 3.1. Data acquisition

All experiments were performed on a 3T-scanner (MAGNETOM Prisma, Siemens Healthcare, Erlangen, Germany) using a 20-channel head and neck coil. The study was approved by the local ethics committee and informed consent was obtained from all volunteers. A prototype pulse sequence was implemented based on a single-shot spin-echo sequence with echoplanar imaging readout that facilitates user-defined gradient waveforms for diffusion encoding (Szczepankiewicz et al., 2019a). Gradient waveforms for spherical b-tensor encoding were numerically optimized (Sjölund et al., 2015), including compensation for concomitant gradients (Szczepankiewicz et al., 2019b). The waveforms were constrained to a maximal gradient magnitude of 80 mT/m (L2-norm) and a slew rate of 100 T/m/s (Appendix B). Detailed information on acquisition parameters will be described per experiment.

Low-resolution data was acquired with slices rotating around a fixed phase encoding direction in the antero-posterior direction. Each FOV covered the entire object with a margin of several voxels, to allow SRR of the entire object. As experiments were performed for various aspect factors, we used the minimum number of low-resolution rotations per aspect factor according to Eq. (1). In simulations, Eq. (2) was used to obtain low-resolution data. In all experiments, low-resolution data was reconstructed per slice to high-resolution according to Eq. (7).

### 3.2. The impact of noise floor on signal accuracy

We characterize the effect of the rectified noise floor on signal accuracy when modulating the aspect factor as well as the strength of diffusion encoding. We use a mono-exponential signal model in water given by

(15) $$S(\alpha ,b)=S_{0}\cdot \alpha \cdot \exp(-b\cdot D),$$

where *S* is the signal in the absence of noise, *S*_0_ is the non-diffusion weighted signal at α = 1, *b* is the strength of the diffusion encoding and *D* is the diffusion coefficient. To obtain a signal model that includes the contribution of Rician noise, we use the simplified analytical model as described in Eq. (8).

Measurements were performed in a phantom filled with deionized water at room temperature. Data were acquired in a single slice with an in-plane resolution of 1.6 × 1.6 mm^2^ and slice thicknesses between 1.6 mm and 9.6 mm, resulting in aspect factors between 1 and 6. A constant TR of 4 s was used to remove influence of T1-relaxation. A single slice acquisition was used to ensure that the same part of the phantom was imaged for all aspect factors, thereby providing a fair comparison of baseline signals. We used b-values ranging from 0 to 3 ms/*μ*m^2^ in steps of 0.3 ms/*μ*m^2^ and 5 repetitions. The average measured signal $\bar{S}(\alpha ,b)$ was estimated for each aspect factor and b-value in a homogeneous area of the phantom. The non-diffusion weighted signal *S*_0_, diffusivity *D* and noise level σ were estimated by a least-squares fit of the measured data to Eq. (8) given Eq. (15)

(16) $${\hat{S}}_{0},\hat{D},\hat{\sigma }=\underset{S_{0},D,\sigma }{\arg\;\min}{\left\|\sqrt{{\left(S_{0}\cdot \alpha \cdot \exp(-b\cdot D)\right)}^{2}+{\left(\sigma \sqrt{\pi /2}\right)}^{2}}-\bar{S}(\alpha ,b)\right\|}_{2}^{2}.$$

The same conditions were reproduced in numerical simulations. The simulations were matched to the observations in the water phantom, and used *S*_0_ = 1 and *D* = 2.2 *μ*m^2^/ms. Instead of using the analytical model for noise contribution, the noise characteristics were simulated by adding noise sampled from a normal distribution with standard deviation σ = 0.014. Independent realizations of noise were added to the real and imaginary part of complex signal generated by Eq. (15), whereafter its magnitude was computed. This yielded an SNR of 71 and 426 for α = 1 and 6, respectively. The average signal $\bar{S}(\alpha ,b)$ was estimated from 10^4^ realizations of noise.

Finally, similar simulations were performed within SRR for aspect factors between 1 and 6, deployed on a digital phantom of a cylinder filled with water (*S*_0_ = 1 and *D* = 2.2 *μ*m^2^/ms). For each aspect factor, a single noisy (σ = 0.014) set of low-resolution measurements was synthesized using Eq. (2) and reconstructed to high-resolution, after which the average signal $\bar{S}(\alpha ,b)$ was computed in a ROI within the cylinder.

The estimate SNFR for each aspect factor, we fitted Eq. (16) to the signal generated by the three methods to obtain σ. From σ and the measured signal $\bar{S}(\alpha ,0)$, we computed SNFR according to Eq. (9). To capture the trend of how SNFR affects the accuracy of signal, we computed the threshold attenuation factor (β_thr_ = *b* · *D*) at which the signal bias exceeds 5%, i.e., $f=\bar{S}(\alpha ,b)/S(\alpha ,b)>1.05$, using

(17) $$\beta_{\text{thr}}=\log\left(\frac{S_{0}\cdot \alpha \cdot \sqrt{f^{2}-1}}{\sigma \sqrt{\pi /2}}\right).$$

The threshold attenuation factors were investigated as a function of aspect factors to visualize how bias thresholds can be extended by imaging at lower resolution.

### 3.3. Analysis of precision and SNR efficiency

We performed numerical simulations to investigate the relationship between SNR efficiency, aspect factor, T1-relaxation and acquisition time. We expect the SNR efficiency to monotonically increase with the aspect factor when TR is relatively long compared to T1. We used a Shepp-Logan phantom to visualize the effect of regularization on resolution. Low-resolution data was sampled for aspect factors between 1 and 8 assuming *S* ∝ α and *S*_α=1_ = 1 in the largest part of the phantom, mimicking white matter. Gaussian noise with σ = 0.1 was added and the SNR efficiency was estimated from the reconstructed images in a homogenous area according to Eq. (11), where we set $\frac{{\text{TA}}_{\text{SRR}}}{{\text{TA}}_{\text{D}}}=\frac{N_{R}}{\alpha }$.

**Fig. C1.**
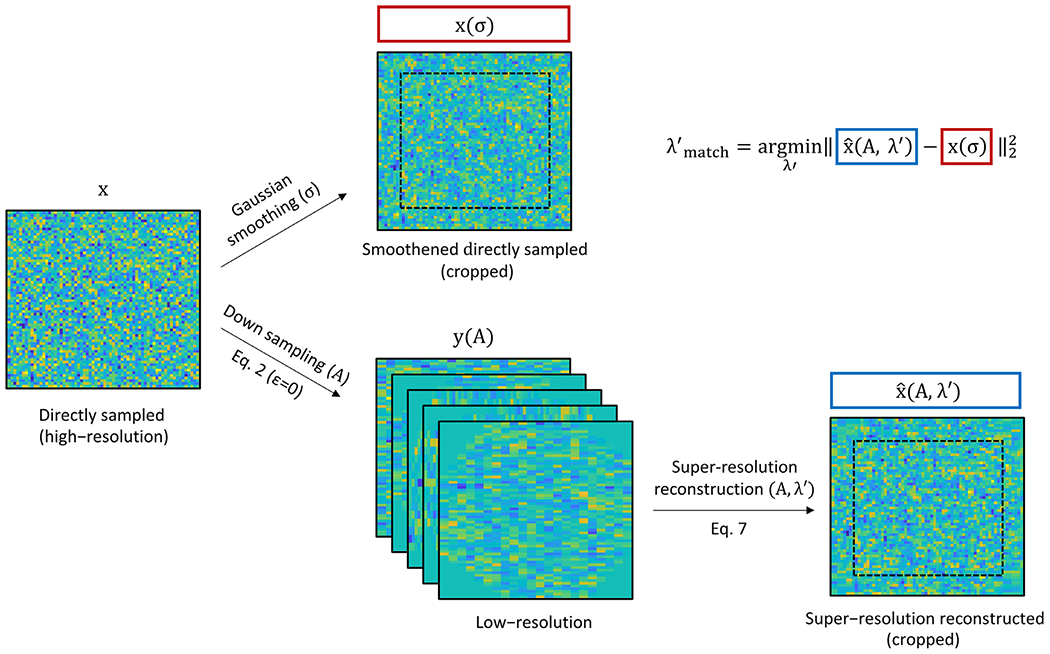
Matching the effective spatial resolution of directly sampled and super-resolution reconstructed images. A noise-free high-resolution image x is smoothened with a given Gaussian kernel size σ and separately down sampled to low-resolution y with a given down sampling matrix A (A is dependent on the aspect factor). The super-resolution reconstructed image $\hat{x}\left(\text{A},\lambda^{\prime }\right)$ has similar effective spatial resolution as the smoothened directly sampled image x(σ) for a regularization strength λ′ that minimizes the sum of squares distance between them. Images are cropped before comparison to avoid influence of edge-artefacts.

Since the regularization in SRR incurs a blurring, it may also cause an unfair advantage in terms of signal precision. To compensate for this effect, we compared SNR efficiency at similar effective spatial resolutions. A graphical overview of this process is given in Appendix C, Fig. C1. Briefly, we ensure similar effective spatial resolutions by matching the effect of smoothing due to regularization in SRR to gaussian smoothing in direct sampling. We base the matching on a noise-free image that depicts a random texture (each voxel value is drawn form a normal distribution). To obtain directly sampled references, this image was smoothened with 2D Gaussian filters with kernel sizes of 0.40 (weak), 0.47 (moderate) and 0.54 (strong) in units of voxels. The SRR counterparts were created by adjusting the regularization strength (λ′ in Eq. (7)) such that the sum of square distances was minimized between the direct and SRR variants. This was done for every combination of kernel size and SRR imaging setup. To avoid the effect of partial SRR sampling at the edges of the FOV, we only matched the region that was covered in all rotations of the FOV. The obtained pairs (smoothing kernel/regularization strength) were used throughout.

The estimation of SNR efficiency was also performed analytically using Eq. (13). As we expect that reducing the TR in SRR leads to reduced SNR efficiency due to T1-relaxation effects, we included T1-weighting in the same equation. We used values of T1 as found in brain at 3T, with T1 = 0.8 s and T1 = 1.6 s to mimic white and gray matter, respectively (Wright et al., 2008). In gray matter, we evaluated SNR efficiency at moderate regularization for TR_D_ = [5 10 20] s. We set TR_SRR_ to the minimum possible value for each aspect factor (Eq. (14)). However, TR_SRR_ does not necessarily have to assume the minimal value and can be increased at the expense of scan time. Increasing TR_SRR_ will lead to an increased SNR efficiency by promoting T1-relaxation, which is counteracted by a decrease in SNR efficiency as the additional time can be used for averaging the direct acquisition (Eq. (13)). Therefore, we investigated how an increased TR_SRR_ affects the SNR efficiency in both gray and white matter for α = 8.

#### 3.3.1. Analysis of precision in vivo

SNR efficiency was evaluated in a healthy brain in vivo (male, 28 years) for aspect factors between 1 and 6. All experiments used *b* = [0 0.5] ms/*μ*m^2^ with 1 and 10 repetitions respectively, FOV = 220 × 220 × 144 mm^3^, TE = 100 ms, partial-Fourier factor = 6/8, an in-plane acceleration factor of 2 (GRAPPA), and bandwidth = 1725 Hz/pixel. Imaging parameters that depended on the aspect factor are summarized in Table 1. All images were reconstructed at a resolution of 1.6 × 1.6 × 1.6 mm^3^. To enable a fair comparison with the direct acquisition approach, it was smoothened using 2D Gaussian with a kernel size of 0.47 to yield an effective spatial resolution similar to a moderately regularized SRR. The SNR efficiency was estimated according to Eq. (11) in the central white matter, brainstem, corpus callosum and cerebellar white matter at *b* = 0.5 ms/*μ*m^2^, whereas images acquired at *b* = 0 ms/*μ*m^2^ are shown.

### 3.4. In vivo dot fraction imaging

We demonstrated the utility of SRR by visualizing the presence of the so-called dot fraction in the brain with a higher contrast than that from direct sampling. The dot fraction is linked to the relative signal that remains at very high b-values when using spherical b-tensor encoding. As anisotropic tissue is effectively attenuated by spherical b-tensor encoding, the remaining signal can be attributed to environments in which the apparent diffusivity is low or zero in all directions (Dhital et al., 2018; Tax et al., 2020).

A healthy volunteer (male, 27 years) was scanned at *b* = [0 1 4] ms/*μ*m^2^ using 1, 3, and 13 repetitions, *N*_*R*_ = 8, resolution = 1.6 × 1.6 × 7.2 mm^3^ (α = 4.5), FOV = 211 × 211 × 144 mm^3^, TE = 120 ms, TR = 4200 ms, TA = 9:31 min, 2x in-plane acceleration (GRAPPA), partial-Fourier = 6/8 and bandwidth = 1720 Hz/pixel. A directly sampled set was acquired for comparison with the same settings, at *b* = [0 1 4] ms/*μ*m^2^ using 1,6, and 33 repetitions, resolution = 1.6 × 1.6 × 1.6 mm^3^, FOV = 211 × 211 × 188 mm^3^, TR = 14,200 ms and TA = 9:28 min. As regularized SRR reduces the spatial resolution slightly, another directly sampled set was acquired with the same settings at a resolution of 1.8 × 1.8 × 1.8 mm^3^ with FOV = 238 × 238 × 162 mm^3^.

The TR was set to the minimum value possible without the use of through-plane acceleration. To reduce the impact of system heating and drift, we used simultaneous multislice acceleration of factor 2 and randomized the b-values across volumes (Hutter et al., 2018; Szczepankiewicz et al., 2021; Vos et al., 2017). All raw data were denoised using Marchenko-Pastur principle component analysis (Cordero-Grande et al., 2019; Tournier et al., 2019). The low-resolution images were reconstructed at a resolution of 1.6 × 1.6 × 1.6 mm^3^ using SRR. A moderate regularization of λ′ = 0.026 was used.

We assume a dot compartment with signal fraction *f*_dot_ and isotropic diffusivity *D*_dot_ equal to zero, accompanied by a fraction of other tissue (1 – *f*_dot_) with non-zero isotropic diffusivity. Assuming Gaussian diffusion and no exchange, the diffusion-weighted signal *S*(*b*) is given by (Dhital et al., 2018; Tax et al., 2020)

(18) $$S(b)=S_{0}\cdot \left(f_{\text{dot}}\;\exp\;\left(-b\cdot D_{\text{dot}}\right)+\left(1-f_{\text{dot}}\right)\;\exp\;\left(-b\cdot D_{\text{other}}\right)\right),$$

where *S*_0_ is the non-diffusion weighted signal. For very high b-values, the exp(−*b*_high_ · *D*_other_ ) ≈ 0, and only signal in the dot compartment remains since *D*_dot_ ≈ 0, which simplifies Eq. (18) and allows us to rewrite to

(19) $$S(b)\approx S_{0}\cdot f_{\text{dot}}\text{ and }f_{\text{dot}}\approx \frac{S(b)}{S_{0}}.$$

The resulting signal map is weighted by both *S*_0_ (T2-weighting) and *f*_dot_ (influenced by density of cells exhibiting restricted diffusion in all directions). Note that, under these assumptions, *f*_dot_ only gives an upper limit on the true value as other tissues as well as the rectified noise floor may contribute to the remaining signal.

Both the super-resolved and directly acquired high-resolution images were averaged for *b* = 4 ms/*μ*m^2^. The gray-to-white matter signal ratio was calculated between gray matter voxels in the cerebellar cortex and white matter voxels in the cerebellum. *f*_dot_ was calculated using Eq. (19). We compared the images to a corresponding T1-weighted morphological scan and to a similar Nissl-stained contrast that emphasize neurons found in brain histology from the BigBrain atlas (Amunts et al., 2013).

## 4. Results

### 4.1. The impact of noise floor on signal accuracy

**Fig. 2.**
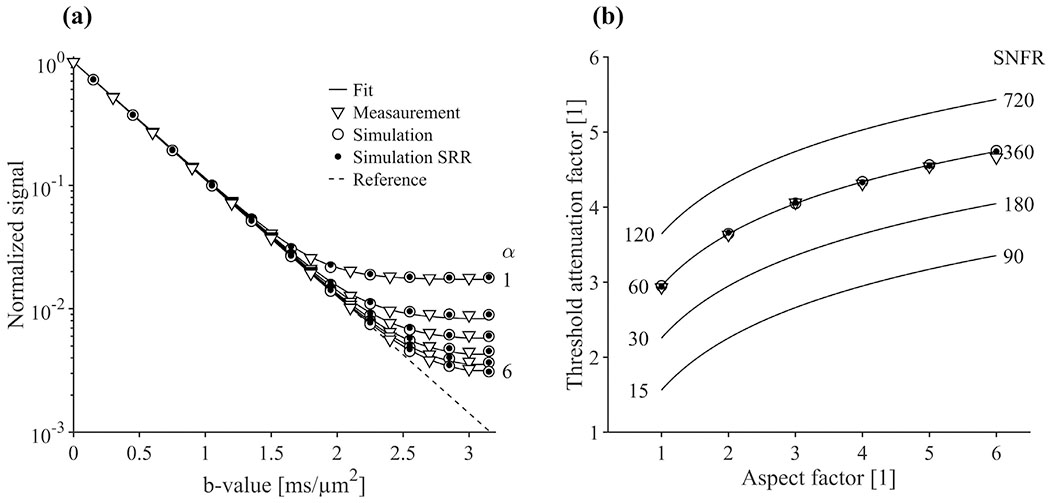
Effect of the rectified noise floor on signal accuracy for aspect factors (α) up to 6. Panel (a) shows the simulated and measured mean signal in a water phantom as a function of the b-value, normalized to signal at b0. Panel (b) shows the threshold attenuation factor where the signal bias caused by the noise floor exceeds 5% versus aspect factor. This is shown for measured SNFR (SNFR 60 at α = 1, SNFR = 360 at α = 6), as well as for various SNRF ranging from 15 for α = 1 to 720 for α = 6. The SNFR grows linearly with voxel size, and the threshold attenuation factor shows similar trends for varying SNFR. Sampling with larger voxels improves accuracy and allows for the use of higher b-values before the noise floor affects the signal. Simulations and experimental results (circles versus triangles) show high agreement.

Fig. 2 shows that the rectified noise floor causes an overestimation of the signal at high b-values where SNR is low (Fig. 2a). The bias can be partially avoided by using higher aspect factors to increase the SNFR. This means that higher b-values can be employed before reaching a given signal bias. In the experiments, the measured signal at α = 1 exhibited an SNFR of 60, which could be increased to 360 at α = 6. By doing so, the maximal b-value could be increased from 1.32 to 2.13 ms/*μ*m^2^ before reaching the 5% signal bias in water at room temperature (Fig. 2b). More generally, increasing the aspect factor from 1 to 6 enabled the attenuation coefficient to become 62% larger (Eq. (17)). Both single voxel and SRR simulations agreed with measurements. Note that TR was here constant across measurements to avoid contributions from T1-weighting.

### 4.2. Analysis of precision and SNR efficiency

**Fig. 3.**
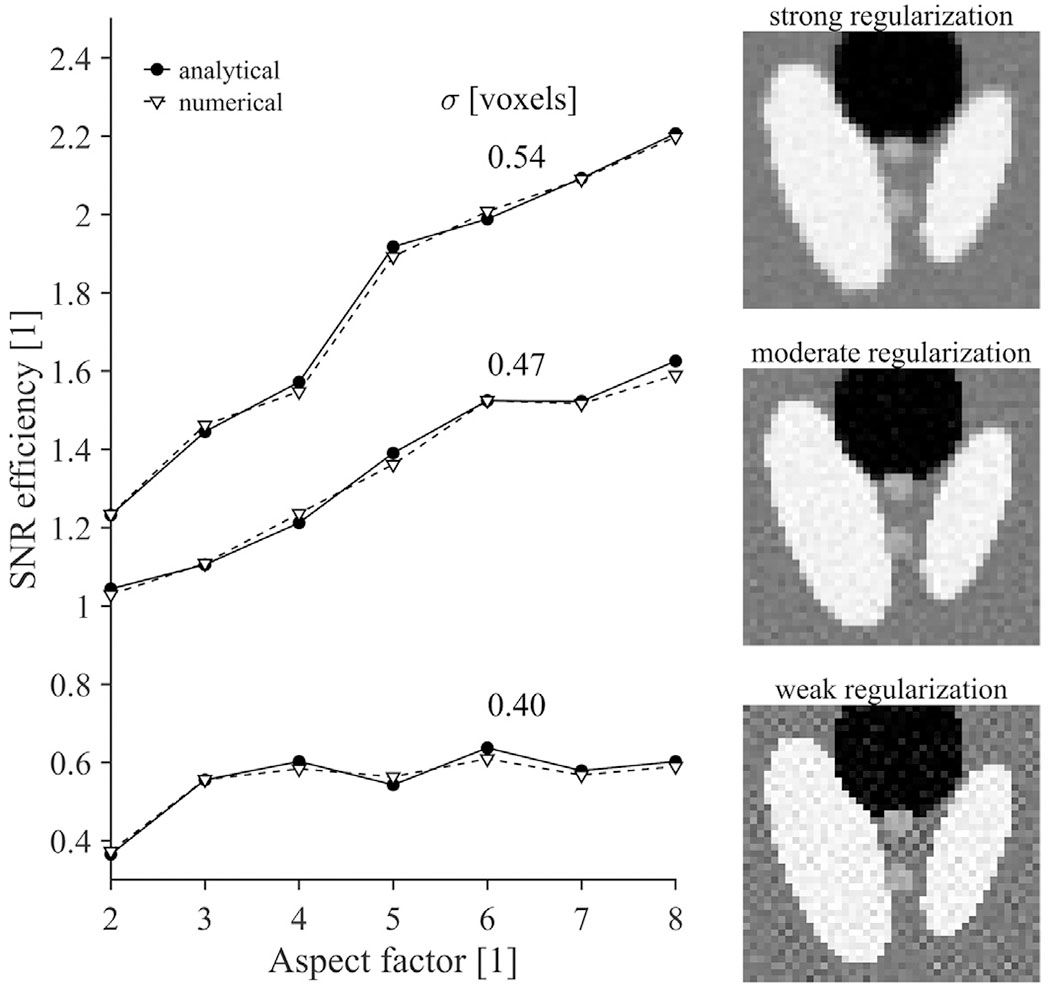
SNR efficiency as a function of aspect factor at multiple regularization strengths, evaluated numerically (dashed line) and analytically (solid line). An SNR efficiency above unity means that precision increases compared to direct sampling with matched spatial resolution and acquisition time. Both the increase in aspect factor and regularization strength lead to a higher SNR efficiency. The magnified Shepp-Logan phantom images (right) show the effect of regularization on image characteristics (spatial resolution/noise propagation), and are reconstructed from 5 low-resolution images with an aspect factor of 3 and a SNR of 30 in the region mimicking white matter. Effects of T1-relaxation were not simulated in this analysis.

**Fig. C2.**
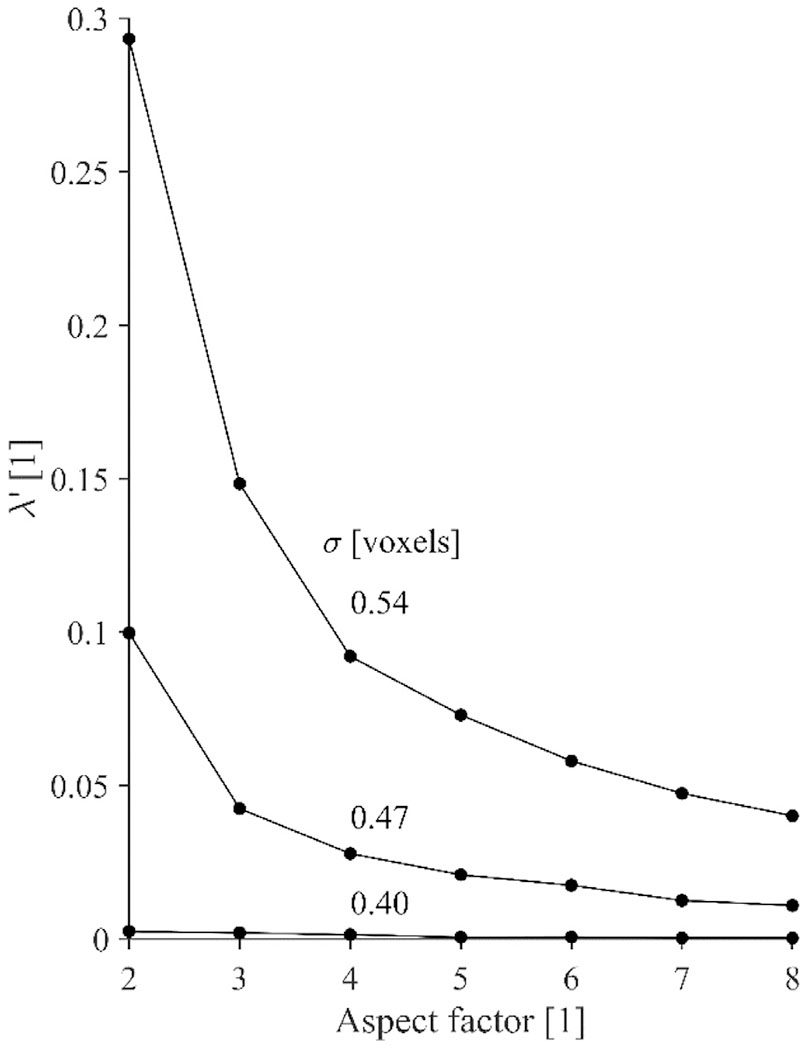
Values of regularization strength λ′ matched with Gaussian kernel sizes σ in terms of induced effective spatial resolution for different aspect factors. λ′ decreases with increasing aspect factor, with a smaller decrease for higher aspect factors. λ′ increases with increasing σ.

Fig. 3 shows the SNR efficiency for SRR with different aspect factors for the three levels of regularization (see Appendix C, Fig. C2 for the regularization values). SNR efficiency generally increases with aspect factor. For example, the SNR efficiency is 1.6 for an aspect factor 8 when using moderate regularization. As expected, strong regularization leads to higher SNR efficiency, but may cause reduced spatial resolution and blurred edges. Conversely, weak regularization may substantially inflate the effects of noise, resulting in SNR efficiency below 1 for all aspect factors. As analytical and numerical results agree, the analytical expression can guide the regularization accurately. Note that this analysis does not include T1-relaxation effects.

**Fig. 4.**
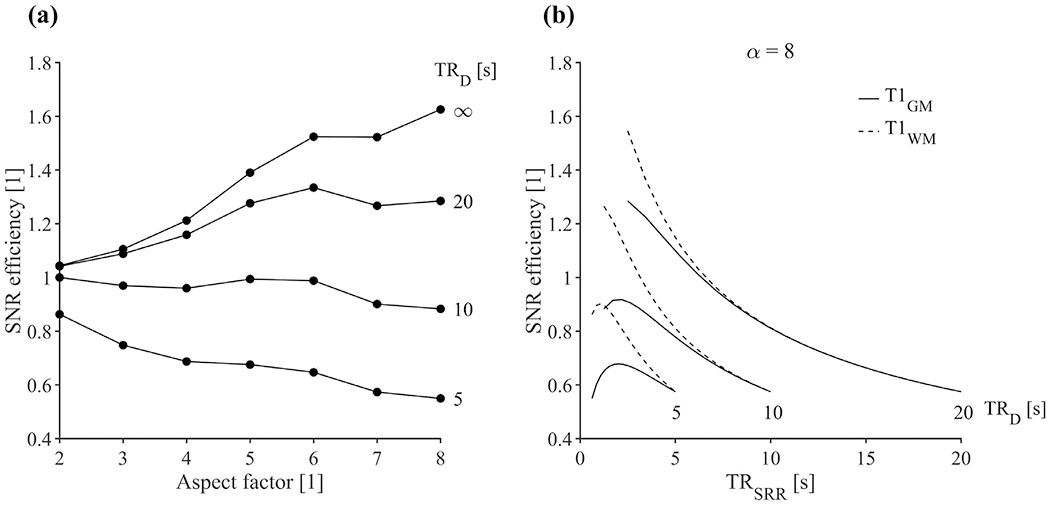
Effect of T1 relaxation on SNR efficiency. Panel (a) shows the SNR efficiency using T1 = 1.6 s and the minimum TR_SRR_ for a given TR_D_ and aspect factor. SNR efficiency decreases as TR_D_ decreases, with a faster decrease for higher α. Panel (b) shows the effect of changing TR_SRR_ above its minimum at the expense of scan time, for *α* = 8 and T1 of 1.6 s and 0.8 s, mimicking gray- and white matter, respectively. SNR efficiency is maximized when TR_SRR_/T1 is as close as possible to 1.25. Simulations were performed with moderate regularization.

Fig. 4 shows the effect of T1-relaxation on SNR efficiency, which decreases with TR_D_ (Fig. 4a). This effect is more evident for higher aspect factors. For example, for aspect factor 8, SNR efficiency reduces from 1.6 to 0.6 as we go from the case where T1-relaxation is neglected (TR_D_ ≫ T1) to a case where TR_D_ = 5 s for a T1 value mimicking gray matter. SRR in this case leads to a reduced SNR efficiency, rather than an increase. However, when TR_D_ ≫ T1, T1-effects are less evident and SNR efficiency is still above unity for SRR. This situation occurs for example when TR_D_ is relatively high and/or low aspect factors are used. Note that TR_SRR_ can be increased to promote T1-relaxation. However, this leads to an increased scan time, which benefits the direct acquisition as this time can be used for signal averaging. As illustrated for an aspect factor of 8, we see it is beneficial to set TR_SRR_ to a minimum when TR_SRR_/T1 > 1.25 (Fig. 4b). Until this point is reached, the benefits of rapid signal-sampling outweigh the benefits of prolonged T1-relaxation in terms of SNR efficiency.

#### 4.2.1. Analysis of precision in vivo

**Fig. 5.**
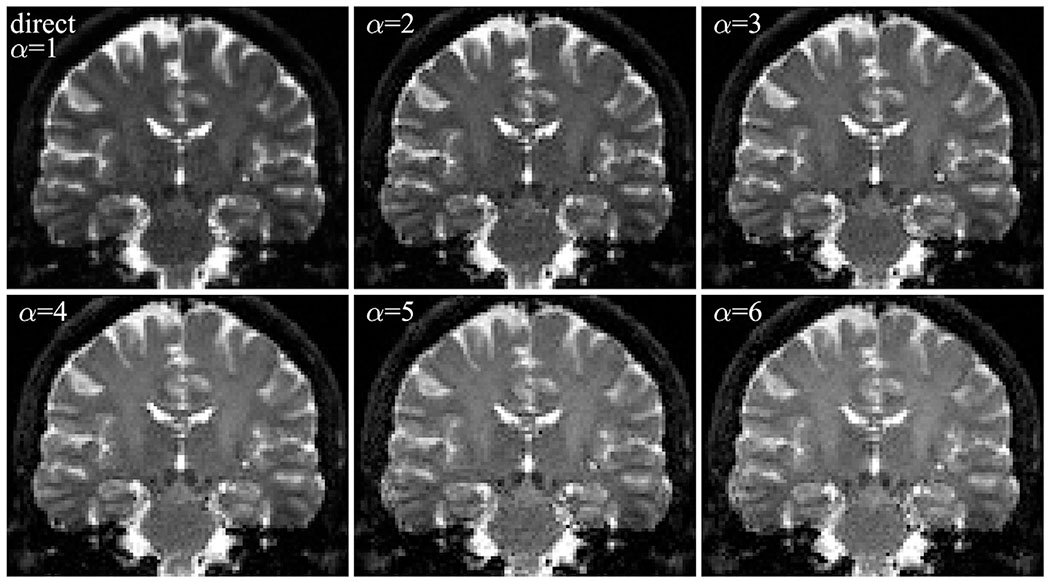
Direct sampling and super-resolution reconstruction (SRR) for various aspect factors (α). Image were obtained in a healthy brain in vivo at *b* = 0 ms/*μ*m^2^ and reconstructed at a resolution of 1.6 × 1.6 × 1.6 mm^3^. All images were reconstructed with moderate regularization and are thus matched in terms of effective resolution (σ = 0.47 voxels), meaning Gaussian smoothing was applied to the direct case. Due to different signal intervals, the maximum of the colormap is scaled separately for each image. Note that the overall image contrast changes with aspect factor due to T1 effects because a higher aspect factor allows a shorter TR. This results in a smaller signal difference between the white matter (T1 = 0.8 s) and the cerebrospinal fluid (T1 = 4.3 s).

Fig. 5 shows the results of SRR in vivo for different aspect factors at similar effective resolution. It shows that SRR in vivo is feasible, as resolution is regained for all aspect factors. Ringing artefacts are observed near high-contrast transitions, such as around the ventricles (Gottlieb et al., 1992). As the aspect factor increases and the TR_SRR_ is reduced, T1-weighting becomes more visible. This can for example be observed in the contrast decrease between white matter and CSF, with T1 of 0.8 s and 4.5 s, respectively (Shin et al., 2009).

**Fig. 6.**
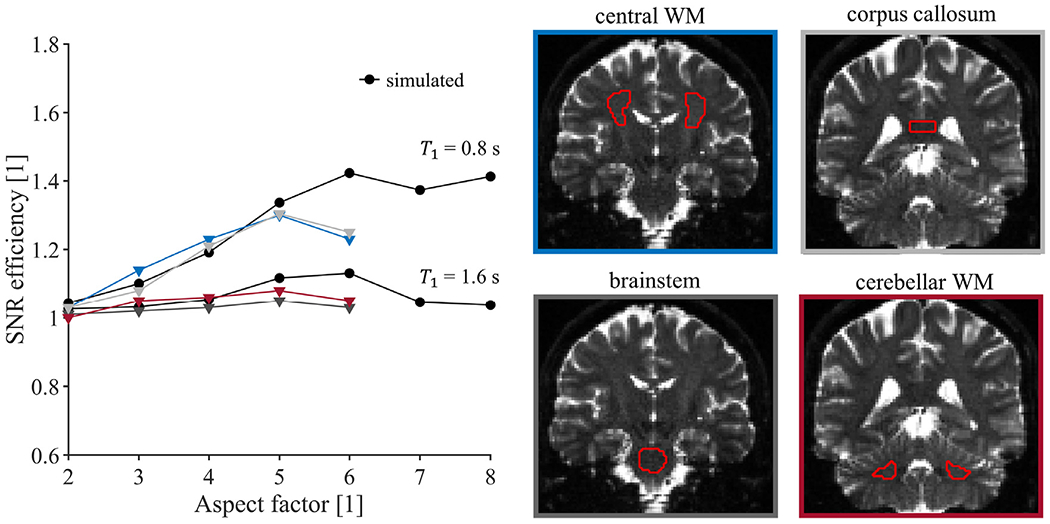
SNR efficiency in vivo. The values were obtained from simulations as well as estimated from experimental data, for different aspect factors. In central white matter and the corpus callosum, trends up to α = 5 are similar to simulations with T1 = 0.8 s, mimicking white matter. The SNR efficiency in brainstem and cerebellum is more similar to simulations with T1 = 1.6 s, mimicking gray matter. Generally, the SNR efficiency in white matter is overestimated by the simulations (black circles) compared with measurements (triangles). Note that SNR efficiency is above unity in all cases.

Fig. 6 shows SNR efficiency for measured and simulated data in white matter at *b* = 0.5 ms/*μ*m^2^. In central white matter and the corpus callosum, experiments and simulations (T1 = 0.8 s) follow similar trends of increased SNR efficiency with aspect factor, up to α = 5. In the brainstem and cerebellar white matter, simulations overestimate the SNR efficiency for all aspect factors and the trends are more similar to simulations with a longer T1 of 1.6 s, mimicking gray matter. A potential explanation for this trend is that ROIs in both brainstem and cerebellum contain gray matter with relatively long T1, thereby reducing SNR efficiency according to Eq. (13). Regardless, the SNR efficiency is still above unity for all cases, meaning that SRR has a higher precision than its directly sampled equivalent for this protocol.

### 4.3. In vivo dot fraction imaging

**Fig. 7.**
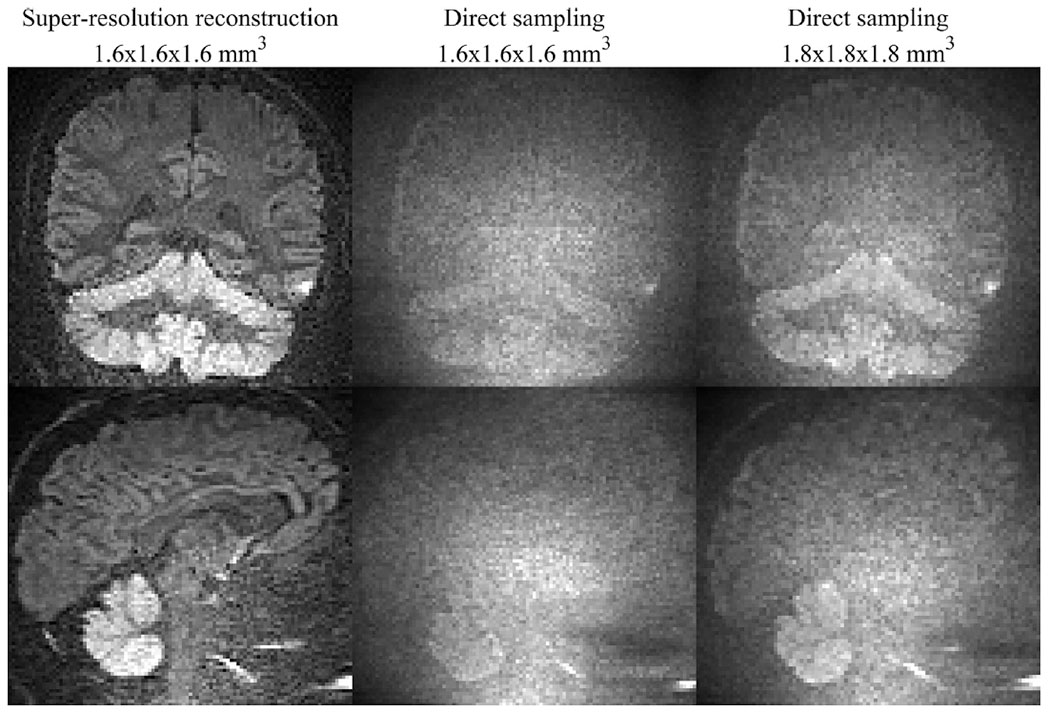
In vivo illustration of the accuracy-related benefit of super-resolution reconstruction (SRR). The figure shows diffusion-weighted images from a SRR protocol (left) and direct acquisition (middle) with spherical encoding at *b* = 4 ms/*μ*m^2^ in coronal and sagittal view at 1.6 × 1.6 × 1.6 mm^3^. As regularization in SRR slightly decreases spatial resolution, we also compare to a direct acquisition at 1.8 × 1.8 × 1.8 mm^3^ (right). A vastly higher contrast is observed with SRR compared with direct sampling at both the target resolution and the slightly reduced resolution. Quantitatively, this corresponds to an increase in the contrast ratio between the cerebellar cortex and white matter to 1.86 from 1.06 (middle) or 1.31 (right). Acquisition times are similar.

**Fig. 8.**
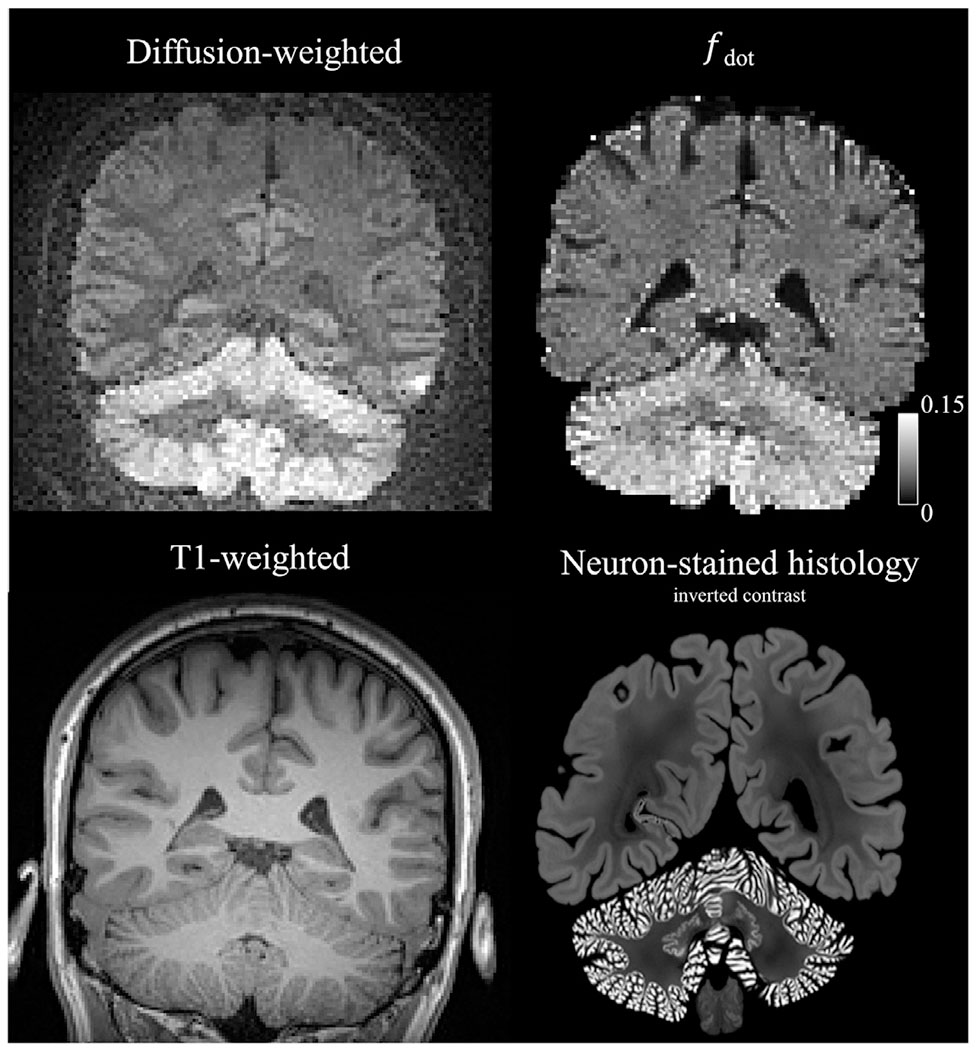
Analysis of the image contrast. The top row shows signal retention using diffusion-weighted imaging with spherical encoding at *b* = 4 ms/*μ*m^2^ (left) and estimation of *f*_dot_ (right). The bottom left panel shows a T1-weighted image of the corresponding slice. The bottom right image shows a neuron-stained histology slice from the human brain, obtained from the BigBrain atlas (Amunts et al., 2013). Regions of high diffusion-weighted signal and high *f*_dot_ are found in the cerebellar cortex where granule cells are densely packed.

Fig. 7 shows results for the dot fraction imaging. Direct sampling at 1.6 mm^3^ isotropic leads to poor image contrast throughout the brain, as the image is dominated by the noise floor. When sampling directly at a resolution of 1.8 mm^3^ isotropic, the cerebellar cortex becomes faintly visible, but the contrast is still poor. On the other hand, SRR enables a vastly improved image contrast where the cortex of the cerebrum is visible with prominent signal. The contrast ratio between cortex and white matter of the cerebellum are 1.86 for SRR versus 1.06 and 1.31 for direct sampling at 1.6 mm^3^ and 1.8 mm^3^ isotropic, respectively. Fig. 8 shows that a part of this contrast is due to T2 effects, as the map of *f*_dot_ shows a less pronounced difference between cerebellar white- and gray matter. A similar contrast is observed in neuron-stained brain histology, where the cerebellar cortex shows high density of neurons.

## 5. Discussion

In this work, we investigated the performance of super-resolution reconstruction and its impact on signal accuracy and precision in diffusion MRI. We found that SRR improved accuracy in a challenging application that would not be feasible without it (Fig. 7). We also expect that the increase in signal accuracy by SRR will lead to an improved accuracy in parameters estimated from dMRI data (Jeurissen et al., 2018).

SRR improves the accuracy of the diffusion-weighted signal by avoiding sampling signal that is biased by the rectified noise floor. This gain is linear with voxel volume in terms of SNFR, which allows the use of stronger diffusion encoding before a signal bias is introduced (Fig. 2). An alternative method to improve accuracy is averaging complex data, which is challenging due to the need of phase correction (Eichner et al., 2015; Prah et al., 2010). The presented methodology uses the magnitude signal, which allows us to avoid this challenge. Although SRR and complex averaging are, in principle, compatible, the investigation of such an approach was outside the scope of this work. Moreover, as signal bias is mostly avoided rather than corrected, SRR does not have to rely on prior knowledge of the data distribution. This differs from postprocessing methods that have been proposed to remove the bias (Andersson, 2008; Kristoffersen, 2007; Veraart et al., 2013, 2011) and can be challenging due to, for example, non-trivial coil-combination methods. An alternative SRR technique is g-slider (Setsompop et al., 2018) where signal is acquired in slabs with different excitation profiles. Although this approach has excellent SNR efficiency, SRR based on rotating FOVs can be deployed with off-the-shelf sequences, whereas g-slider requires a specialized pulse sequence. Nevertheless, a combination of g-slider, spherical b-tensor encoding and high spatial resolution may enable even better resolution, contrast and/or shorter acquisition times.

We found that SNR efficiency can be preserved or even improved using SRR, depending on the aspect factor, T1-weighting, and regularization. The established analytical formulation for SNR efficiency (Eq. (13)) may help setting the regularization strength to decide the tradeoff between SNR efficiency and effective spatial resolution (Fig. 3). Simulations show that SNR efficiency is generally increased for large aspect factors, however, effects of T1-relaxation become dominant at sufficiently high aspect factors (Fig. 4). Previous studies suggested larger aspect factor would lead to larger improvements in SNR efficiency (Van Steenkiste et al., 2016). However, this was under the assumption of complete T1-recovery. The effects of T1-relaxation counteract the SRR improvement when relatively short repetition times are used, which has become more common since the simultaneous multi-slice techniques were made available (Barth et al., 2016). Furthermore, the use of thicker slices increases sensitivity to motion-induced gradient imbalances leading to intravoxel dephasing and therefore loss of signal, which may partially explain the mismatch between simulations and in vivo results (Fig. 6) and put a practical limit on the maximum achievable aspect factor (Gumus et al., 2014). Generally, we see that the potential precision increase is heavily dependent on acquisition, reconstruction, and intrinsic tissue parameters.

The gain in precision from SRR was only modest, while accuracy can be substantially improved by SRR in low SNR scenarios. In practice, this leads to an improved contrast, as the use of larger voxels will reduces the influence from the noise floor and therefore allow for a better differentiation of true signal differences. We demonstrated this in vivo for so called dot-fraction imaging, which visualizes densely packed cells located in the cerebellar cortex and was first demonstrated on a custom MRI system with ultra-strong gradients and at a poor spatial resolution (Tax et al., 2020). Using SRR, we enabled high-resolution imaging at 1.6 mm^3^ isotropic at *b* = 4 ms/μm^2^ with spherical tensor encoding, where the elevated SNFR causes contrast to be substantially improved compared to direct sampling (Fig. 7). If a linear relationship with voxel volume is assumed, the same SNFR is expected for direct sampling only at 2.7 mm^3^ isotropic. We believe that this approach facilitates high-resolution studies of neurodegenerative diseases where isotropic tissues are affected. Current acquisition times are just below 10 min, and the sampling scheme can be further optimized to comply with clinical routine.

Under high-SNR conditions, the simplicity of direct sampling may outweigh the benefits of SRR. However, a remaining benefit of SRR is that it may be used for acceleration when used in combination with a diffusion model (Jeurissen et al., 2018; Van Steenkiste et al., 2016). Acceleration can be achieved by subsampling the diffusion encoding directions or b-tensors over the acquired low-resolution images, such that directional information needed to fit the model can be acquired in a shorter time.

We have identified five primary limitations of the present study. First, to compare regularized SRR and direct sampling at similar effective resolution, we matched the effect of regularization strength and Gaussian smoothing kernel size. However, matching spatial resolutions across filtering and/or reconstruction methods remains a challenge as metrics of spatial resolution are lacking (den Dekker and van den Bos, 1997). Notably, both regularization and smoothing always lead to an effective resolution that is lower than the original or nominal resolution. As such, sampling directly at the effective resolution would increase both SNFR (Fig. 2) and SNR efficiency (Scouten et al., 2006) compared to sampling at the original resolution and performing post-hoc smoothing. For an evaluation of effective spatial resolution and therefore better assessment of SRR, suitable phantoms could be used in future work (Fellner et al., 2001; Price et al., 1990). Second, our analysis does not include motion and eddy current correction in the SRR model, while perfect registration in SRR is of importance to obtain non-blurry high-resolution results. Including such corrections could improve the sharpness of in vivo results. Third, we used the identity matrix as the regularization matrix (Eq. (7)). Using data-driven regularization that depends on image content can have benefits, such as edge-preservation (Khattab et al., 2020). Forth, the multi-stack rotation SRR model suffers from signal over- and undershoot problems at contrast-rich regions due to regularization, as more thoroughly described by (Shilling et al., 2009). This could be a confounder in quantitative measures extracted from the images, for example when estimating the dot-fraction. Fifth, while concomitant gradients are compensated in the diffusion encoding (Szczepankiewicz et al., 2019b), the read-out gradients are rotated along with the FOV and their concomitant gradients may cause rotation-dependent artefacts (Du et al., 2002).

In conclusion, we have presented a comprehensive analysis of SRR that outlined the major features influencing the precision and accuracy of the diffusion-weighted signal. The primary benefit of SRR is the improved signal-to-noise-floor ratio, but precision can also be improved given beneficial imaging conditions. We showcased the use of SRR in a challenging combination of high resolution and spherical tensor encoding with ultra-high b-values, where SRR can suppress noise floor effects and recover high signal accuracy. We share the tools developed herein in open-source and expect that the present work will support future experimental design, such that both acquisition and reconstruction parameters can be optimized for specialized purposes.

## Figures and Tables

**Table 1:** in vivo acquisition and reconstruction parameters for super-resolution reconstruction protocols. Note that the three numbers that describe the spatial resolution (Res) state the in-plane resolution first, whereas the final value is the slice thickness.

	Direct, α=1	α=2	α=3	α=4	α=5	α=6
*N*_*R*_ [1]	1	3	5	7	8	10
Res [mm^3^]	1.6 × 1.6 × 1.6	1.6 × 1.6 × 3.2	1.6 × 1.6 × 4.8	1.6 × 1.6 × 6.4	1.6 × 1.6 × 8.0	1.6 × 1.6 × 9.6
TR [ms]	13,000	6500	4300	3300	2600	2200
TA [min]	2:23	3:35	4:00	4:14	3:49	4:02
λ′ [1]	–	0.100	0.043	0.028	0.021	0.018

**Fig. B1.**
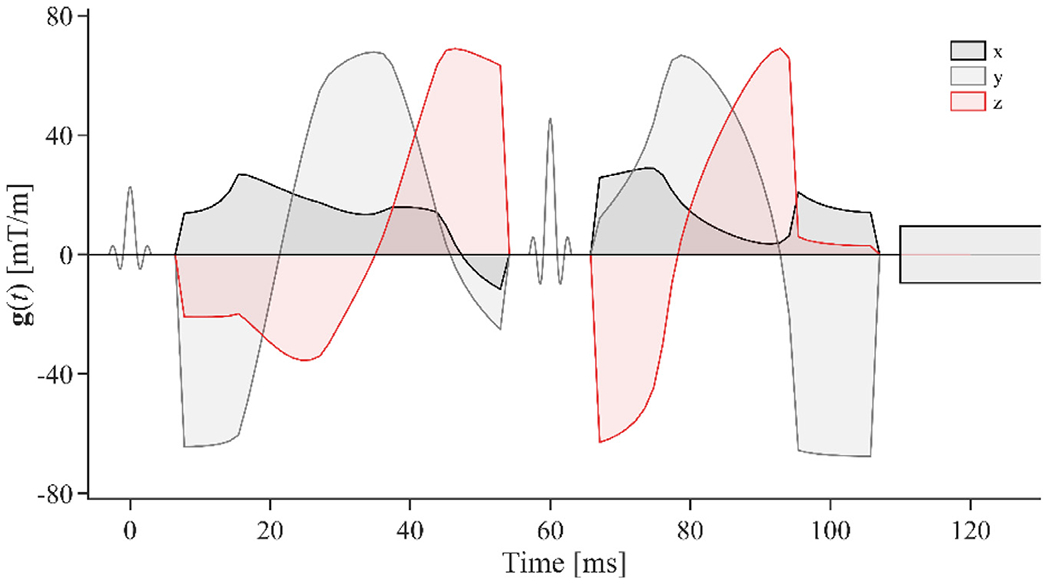
Optimized gradient waveforms were used to yield spherical tensor encoding (STE) in all practical experiments, and the one used for in vivo measurements is shown under conditions that yield *b* = 4.0 ms/μm^2^. Radiofrequency pulses (RF) are depicted in between gradients. This waveform and other resources related to the free waveforms sequence are available at https://github.com/filip-szczepankiewicz/fwf_seq_resources.

## Data Availability

The data that support the findings of this study are not available due to ethical restrictions. The super-resolution reconstruction framework is available in open source at https://github.com/filip-szczepankiewicz/Vis_NIMG_2021/.

## Footnotes

1 We use the terminology where accuracy is only a description of systematic errors. This is referred to as ‘trueness’ in the more recent ISO standard (BS ISO 5725-1, 1994).

## Appendix A. Derivation of SNR efficiency

We derive ρ as given in Eq. (13). We start from Eq. (11), stating

(A1)
$$\rho =\frac{{\text{SNR}}_{\text{SRR}}}{{\text{SNR}}_{\text{D}}}\cdot \sqrt{\frac{{\text{TA}}_{\text{D}}}{{\text{TA}}_{\text{SRR}}}}.$$

To find our final expression, we express both SNR_SRR_ and SNR_D_ in terms of SNR_LR_, the SNR of a low-resolution acquisition. For all cases,

(A2)
$${\bar{\text{S}}}_{x}\propto V_{x}\cdot \left(1-\exp\left(-{\text{TR}}_{\text{x}}/\text{T}1\right)\right),$$

where $\bar{S}$ is the mean signal, *V* is the voxel size and *x* the reflecting component, i.e. LR, SRR or D (direct sampling). As *V*_LR_ = α · *V*_D_, we rewrite Eq. (A2) to

(A3)
$${\bar{S}}_{\text{D}}=\frac{{\bar{S}}_{\text{LR}}}{\alpha }\cdot \frac{1-\exp\left(-{\text{TR}}_{\text{D}}/\text{T}1\right)}{1-\exp\left(-{\text{TR}}_{\text{LR}}/\text{T}1\right)}.$$

As σ is independent of voxel size, σ_D_ = σ_LR_ (Edelstein et al., 1986), and combining both Eq. (A3) and Eq. (10) gives

(A4)
$${\text{SNR}}_{\text{D}}=\frac{1}{\alpha }\cdot \frac{1-\exp\left(-{\text{TR}}_{\text{D}}/\text{T}1\right)}{1-\exp\left(-{\text{TR}}_{\text{LR}}/\text{T}1\right)}\cdot {\text{SNR}}_{\text{LR}}.$$

Along the same way we can find an expression for SNR_SRR_. As TR_SRR_ = TR_LR_, T1-effects are the same for both measurements, and we use Eq. (A2) to see that

(A5)
$${\bar{S}}_{\text{SRR}}=\frac{{\bar{S}}_{\text{LR}}}{\alpha }.$$

We use the definition of the noise propagation factor *κ* of Eq. (12) and Eq. (10) to find

(A6)
$${\text{SNR}}_{\text{SRR}}=\frac{1}{\alpha \cdot \kappa }\cdot {\text{SNR}}_{\text{LR}}.$$

As for SRR the repetition time decreases, but the number of slice orientations increases with a factor *N*_*R*_, we find

(A7)
$$\frac{{\text{TA}}_{\text{SRR}}}{{\text{TA}}_{\text{D}}}=\frac{N_{R}\cdot {\text{TR}}_{\text{SRR}}}{{\text{TR}}_{\text{D}}}.$$

Combing Eq. (A4), Eq. (A6) and Eq. (A7) in Eq. (A1), gives the SNR efficiency ρ defined in Eq. (13), being

(A8)
$$\rho =\frac{1}{\kappa }\cdot \frac{1-\exp\left(-{\text{TR}}_{\text{SRR}}/\text{T}1\right)}{1-\exp\left(-{\text{TR}}_{\text{D}}/\text{T}1\right)}\cdot \sqrt{\frac{{\text{TR}}_{\text{D}}}{N_{R}\cdot {\text{TR}}_{\text{SRR}}}}.$$

## Appendix B. Gradient waveforms

Conventional, or linear diffusion encoding yields a pair of trapezoidal pulsed field gradient on each side of the refocusing pulse in a spin-echo sequence (Stejskal and Tanner, 1965). In this work, we used more advanced, spherical encoding where all three gradients are continuously used (Szczepankiewicz et al., 2019a). The gradient waveforms we used are depicted in Fig. B1.

## Appendix C. Matching the effective spatial resolution

The effective spatial resolution between super-resolution reconstructed and directly sampled images are matched, as illustrated in Fig. C1. The regularization values used for different aspect factors are given in Fig. C2.
